# Spontaneous pneumomediastinum in A term newborn: atypical radiographic and ct appearances

**DOI:** 10.1259/bjrcr.20180081

**Published:** 2019-11-15

**Authors:** Maria Raissaki, Emanouella Modatsou, Eleftheria Hatzidaki

**Affiliations:** 1 Assistant Professor in Pediatric Radiology, Radiology Department, University Hospital of Heraklion, University of Crete, Greece; 2 Senior Registrar, Radiology Department, University Hospital of Heraklion, University of Crete, Greece; 3 Assistant Professor in Neonatology, Department of Neonatology, University Hospital of Heraklion, University of Crete, Greece

## Abstract

Spontaneous pneumomediastinum in the term newborn is relatively rare. We aim to emphasize the significance of Radiographic and CT features of spontaneous pneumomediastinum. We present an otherwise healthy 4-hour-old male, born by a caesarean section, presenting with sudden bulging of the left hemithorax and moderate respiratory distress. Chest radiography revealed an atypical unilateral lobular lucency distorting the mediastinum. CT scan showed an air collection with a multilobular upper border, containing thin straight and curvilinear septa. All segmental bronchi were identifiable. Limited scanning in decubitus position confirmed displacement of thymus and mediastinal air. Following conservative treatment, radiographic findings and symptoms resolved. Pneumomediastinum in neonates, unlike adults, loculates and does not dissect around broncho-vascular structures. Internal septa represent fascia surrounding the thymus. Thymic elevation and absence of lung abnormality constitute helpful imaging findings in support of spontaneous pneumomediastinum so that unnecessary and potentially harmful thoracotomy or chest tube placement are confidently avoided.

## Clinical presentation and differential diagnosis

**Figure 1.**
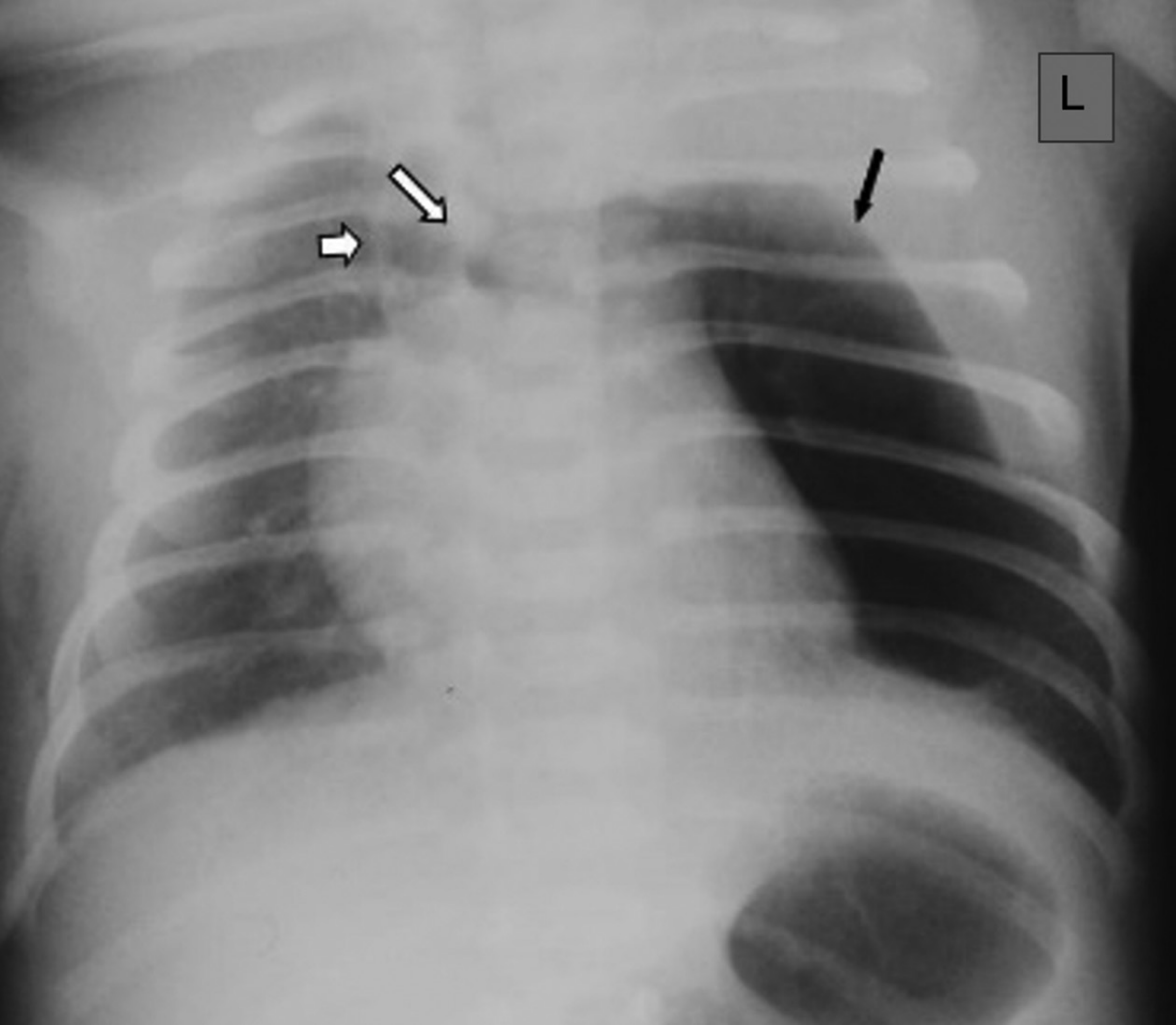
Chest radiograph showing a hyperlucent left hemithorax due to a median lobular air collection, displacing and distorting the mediastinum. A solid density at the left thoracic apex, displaying a convex inferior border (black arrow), suggested a displaced thymus but also mimicked an apical cap. Two thin short radiopaque vertical lines were noted at the sternal area, one midline (long arrow) and one to the right of the mid line (short arrow), reminiscent of anterior junction lines or septa. Note the indirect signs of increased volume of the thoracic cavity on the left with splaying of the ribs ipsilaterally.

A baby male was born at 38-week’s gestation by an uncomplicated caesarean section, he weighed 3240 g, and did not require postnatal resuscitation. Apgar score was 9 at the first and 10﻿^th^ minute following delivery. One hour later, he developed respiratory distress with grunting and tachypnea. Following admission to the neonatal unit, reduction of respiratory sounds and sudden anterior bulging were noted over the left hemithorax. Chest radiography 3 h later showed an atypical air collection (Figure 1). Differential diagnosis included a mediastinal or a medially loculated pleural air collection and, less likely, an intrapulmonary cystic lesion.

## Further investigations

**Figure 2.**
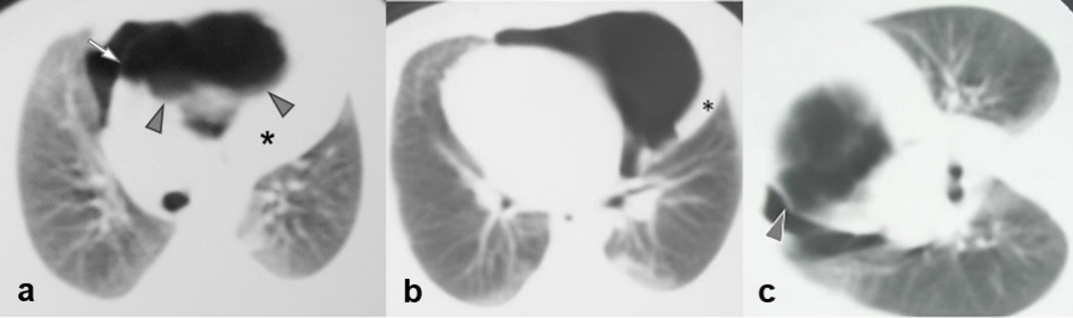
CT. a, b: Transverse scan through the thoracic apex and lower pulmonary hila, respectively. An air collection with a multilobular upper border (arrowheads) is located anteriorly and mostly to the left of the midline, compressing adjacent mediastinal structures, displacing the thymus to the left and the large vessels and heart to the right. The triangular density on the chest radiograph corresponded to an elevated and displaced thymus (*). The air collection contains a thin septum (arrow) that corresponds to the midline short vertical line on the radiograph. The vertical line to the right corresponds to the right edge of the air collection neighboring the displaced right lung, slightly crossing the midline. Note the haziness of lung parenchyma at the subpleural areas posteriorly and a small subpleural nodule on the left. C. Limited CT scan of the mid thorax with the patient at the right decubitus position. The size and shape of the air collection remained stable, indicating a closed air collection and excluding a pneumothorax. Note again the multilobular upper border of the mediastinal air collection containing very thin septae and a relatively thicker central septum (arrowhead).

Two hours later, the neonate underwent CT while being under oxygen therapy through a hood. A lateral decubitus radiograph or an ultrasound were not requested. CT determined the position and configuration of air and its relationship with adjacent structures (Figure 2). Limited CT scan with the baby at the right decubitus position showed a stable air collection in support of its mediastinal location. Identification of all segmental bronchi of the left lung was a finding which supported further the extrapulmonary location of air. Patchy subpleural areas of ground glass were found posteriorly, also forming small nodules, and were attributed to dependency changes under the context of wet lung or aspiration.

## Treatment and outcome

**Figure 3.**
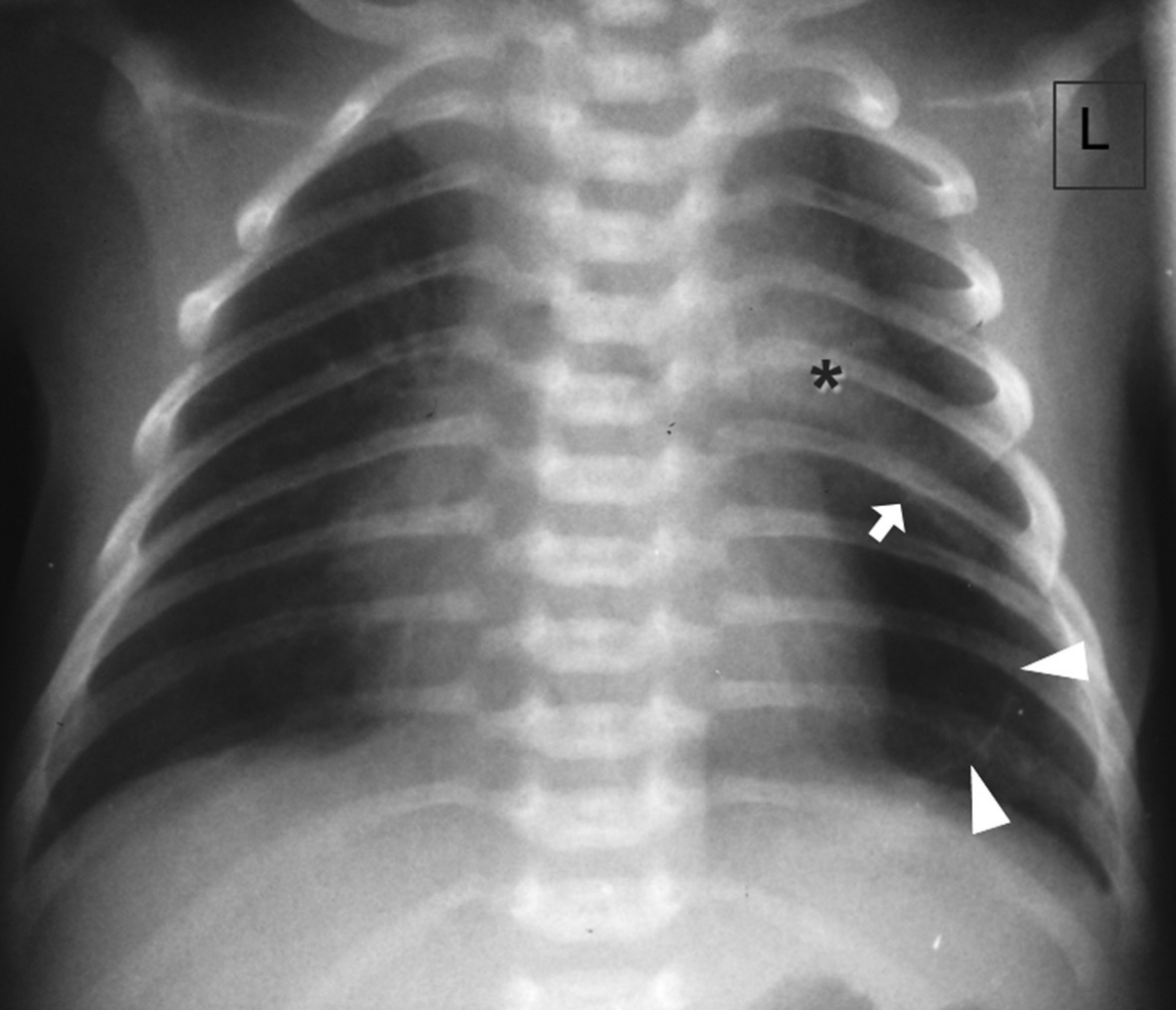
Chest radiograph at the age of 5 days showing a small median loculated air collection, outlining the left heart border and typically the convex inferior border (arrow) of a non-displaced thymus (*). Note a thin line (arrowheads) outlining the lateral aspect of residual pneumomediastinum, thought to represent the combination of displaced pleura with areolar tissue which connects the thymic capsule with the pericardium.

Findings strongly favored the diagnosis of spontaneous pneumomediastinum. The patient remained under oxygen therapy through a hood and was closely monitored. During the following 4 days, symptoms gradually resolved. A chest radiograph on the fifth day showed improvement and more typical radiographic appearances (Figure 3). Final diagnosis was spontaneous pneumomediastinum. The patient was discharged in good condition. Follow-up 8 months later confirmed normal development.

## Discussion

Neonatal pneumomediastinum occurs in approximately 2.5 per 1000 live births and in 0.1% of neonates hospitalized in intensive care units.^1,2^ Most cases of pneumomediastinum are associated with prematurity and treatment for surfactant deficiency while in term newborns predisposing factors include meconium aspiration syndrome, pneumonia, labor-related trauma, as well as positive pressure during resuscitation or mechanical ventilation.^1–6^

Spontaneous pneumomediastinum is a non-traumatic mediastinal air-leak without an underlying lung disease.^3–5^ The diagnosis of spontaneous pneumomediastinum is usually unsuspected without imaging.^3–5^ Pneumomediastinum typically appears as radiolucent air collections outlining the thymus.^5^ In cases of atypical radiographic appearances, CT may further characterize radiolucencies which are located medially or posteriorly.^6^ The multiloculated CT appearance of pneumomediastinum has been previously mentioned in newborns.^4,5,7^ We are describing the clinical, atypical radiographic and CT features of spontaneous pneumomediastinum in a term newborn and discuss the role of CT in the differential diagnosis and management of difficult cases.

In the ventilated newborn, pneumomediastinum occurs following rupture of overdistended immature or diseased alveoli. Spontaneous pneumomediastinum in otherwise healthy term newborns is rare and underdeveloped alveoli have been incriminated for its development.^4,8^ Air dissects in a centripetal fashion into the perivascular and peribronchial fascial sheaths and centrally into the areolar tissue of the mediastinum.^9^ Uneven expansion of pulmonary segments due to aspirated material may lead to a ball valve effect within the bronchus, increased pressure locally and alveolar rupture.^8^ Aspiration of amniotic fluid, blood or meconium has been associated with cesarean section, with subsequent development of respiratory distress and malignant pneumomediastinum.^10^ In our case, there were no oligohydramnios, prematurity, prior assisted ventilation, the patient was delivered uneventfully while small subpleural nodules and ground glass could be attributed to dependency changes, residual retained fluid or previous aspiration; therefore, minimal aspiration during delivery might have caused the air-leak.

Pneumomediastinum is a benign, self-limiting neonatal condition, it may have a symptomatic or asymptomatic course and resolves with supportive conservative treatment.^6,8,10^ Criteria for malignant pneumomediastinum requiring intermittent aspiration and/or mediastinostomy include radiographically confirmed pneumomediastinum, deteriorating oxygen saturation and increasing metabolic acidosis.^11^ Accurate and prompt clinical and imaging diagnosis of neonatal pneumomediastinum is important for patient’s management.

Classic radiographic signs include air elevating the thymus and giving rise to a crescentic configuration akin to a windblown spinnaker sail.^4,5^ When gas is interposed between the heart and the diaphragm, the central portion of the diaphragm which is usually unidentifiable becomes visible, resulting in the “continuous diaphragm” sign.^9,10^ Atypical appearances may occur, especially when air is situated at the lower half of the thorax and seen as loculated midline air collections posterior to the heart, an appearance known as infra-azygous pneumomediastinum.^10^ In our case relatively unusual radiographic features included unilateral location of air, obliteration of the upper surface of the thymus against the thoracic apex mimicking an apical cap and associated space-occupying phenomena with potential large vessels compression. Radiographic appearances of spontaneous pneumomediastinum may be confusing, and the diagnosis may prove difficult especially when one is unfamiliar with the entity.^5,7,10,11^

In neonatal pneumomediastinum, a thin line may be seen connecting the inferior pole of the displaced thymus to the midportion of the heart, with or without tenting of the pericardium at the point of attachment. This line corresponds to connective tissue forming a fascia that envelopes the thymus and merges with the fibrous pericardium.^3,7^ Gas dissecting into the interlobular septa of the thymus that are thought to be contiguous with the thymic capsule, may result in one or more visible septa.^4^ Therefore, thin radiopaque lines at the lateral aspect or within mediastinal air collections should not be mistaken for junction lines which are encountered in radiographs of adults nor for coexisting pneumothorax with pneumomediastinum. Limited CT scanning at decubitus position was useful in our case, because the consideration of a medial pneumothorax requiring drainage was promptly and safely excluded. We are in no position to comment on the utility of a lateral decubitus radiograph or an ultrasound scan since they were not performed.

The differential diagnosis of spontaneous, medially located, loculated air collections extending to the periphery of the lung field on a radiograph includes pneumothorax, pneumomediastinum, pneumopericardium and intrapulmonary lesions such as cystic pulmonary adenomatoid malformation, congenital lobar emphysema and spontaneous unilateral localized pulmonary interstitial emphysema. The distribution of air on a supine radiograph with or without an additional decubitus radiograph may determine the position of air. Congenital gas-containing lesions are usually accompanied by an appropriate antenatal diagnosis and/or deteriorating symptoms. Pulmonary interstitial emphysema (PIE) is best visualized by CT and appears as meandering tubular and cystic lucencies that fail to conform to the predictable branching pattern of air bronchograms, contain a characteristic linear and dot-like structure of soft-tissue attenuation which corresponds to bronchovascular bundles.^12^

Ultrasonography of the mediastinum with the transducer placed directly over the anterior chest wall has also been utilized.^13^ Ultrasound may demonstrate air as thick echogenic lines along the anterior or lateral margins of the thymus, between the posterior margin of the thymus and great vessels or within the thymus parenchyma.^13,14^ Air in the infra-azygous space or inferior pulmonary ligament may be ultrasonographically missed.^15^

## Teaching points

- Spontaneous neonatal pneumomediastinum may prove to be a challenging diagnosis.
- Although a loculated radiolucency distorting the mediastinum and elevating the thymus on a chest radiograph is considered typical of pneumomediastinum, the entity may present with a large, confusing unilateral air collection.
- Septa within neonatal pneumomediastinum correspond to areolar connective tissue which connects the thymic fascia to the pericardium.
- A false diagnosis of anterior pneumothorax could erroneously lead to inappropriate and potentially harmful placement of a drainage tube.
- When in doubt, a lateral decubitus radiograph, Ultrasound with the neonate in various positions with various probes and lastly CT may prove useful.
- Visibility of all segmental bronchi in compressed lung parenchyma and lack of an antenatally diagnosed pulmonary lesion exclude an intrapulmonary air-containing condition requiring thoracotomy.

## Informed consent

Written informed consent for the case to be published (including images, case history and data) was obtained from the guardians for publication of this case report.
